# Case of Lithotomy; the Stone Situated behind the Os Pubis

**Published:** 1829-09

**Authors:** T. Hodson

**Affiliations:** Surgeon.


### LITHOTOMY.

### *Case of Lithotomy; the Stone situated behind the Os Pubis.*

By T. Hodson, Esq. Surgeon.

Having read Mr. Wickham's case of lithotomy in this Journal for February last, I am induced to send you the following case, which, although the operation was finished in the course of a few minutes, might have proved very tedious and distressing both to myself and the patient.

Upon cutting into the bladder, and introducing the fore-finger of my left hand upon the cutting gorget, I perceived the stone behind the os pubis, and so situated that it would have been impossible to have taken hold of it with the straight forceps. I therefore immediately introduced the curved forceps upon the blunt gorget, and readily got hold of the stone; but, upon attempting to bring it down, the forceps slipped and let go their hold. This happened four or five times before I could succeed in bringing it down, when it was easily extracted, and the patient, who was about forty years of age, recovered.

The stone weighed three ounces, and was shaped like a heart, and, being situated behind the os pubis, with its apex downwards, the difficulty alluded to was readily explained; and, although the operation (as I have already observed) was finished in the course of a few minutes, it might, I conceive, have proved very tedious and embarrassing, in consequence of the stone being wedged in the arch of the pubis.

*Lewes; June*, 1829.

